# The STK33-Linked SNP rs4929949 Is Associated with Obesity and BMI in Two Independent Cohorts of Swedish and Greek Children

**DOI:** 10.1371/journal.pone.0071353

**Published:** 2013-08-15

**Authors:** Mathias Rask-Andersen, George Moschonis, George P. Chrousos, Claude Marcus, George V. Dedoussis, Robert Fredriksson, Helgi B. Schiöth

**Affiliations:** 1 Department of Neuroscience, Functional Pharmacology, Uppsala University BMC, Uppsala, Sweden; 2 Department of Nutrition and Dietetics, Harokopio University of Athens, Athens, Greece; 3 Department for Clinical Science Intervention and Technology (CLINTEC), Department of Pediatrics, Karolinska University Hospital Karolinska Institutet, Huddinge, Sweden; 4 First Department of Pediatrics, Athens University Medical School, Aghia Sophia Children’s Hospital, Athens, Greece; Sanjay Gandhi Medical Institute, India

## Abstract

Recent genome wide association studies (GWAS) have identified a locus on chromosome 11p15.5, closely associated with serine/threonine kinase 33 (STK33), to be associated with body mass. STK33, a relatively understudied protein, has been linked to KRAS mutation-driven cancers and explored as a potential antineoplastic drug target. The strongest association with body mass observed at this loci in GWAS was rs4929949, a single nucleotide polymorphism located within intron 1 of the gene encoding STK33. The functional implications of rs4929949 or related variants have not been explored as of yet. We have genotyped rs4929949 in two cohorts, an obesity case-control cohort of 991 Swedish children, and a cross-sectional cohort of 2308 Greek school children. We found that the minor allele of rs4929949 was associated with obesity in the cohort of Swedish children and adolescents (OR = 1.199 (95%CI: 1.002–1.434), p = 0.047), and with body mass in the cross-sectional cohort of Greek children (*β* = 0.08147 (95% CI: 0.1345–0.1618), p = 0.021). We observe the effects of rs4929949 on body mass to be detectable already at adolescence. Subsequent analysis did not detect any association of rs4929949 to phenotypic measurements describing body adiposity or to metabolic factors such as insulin levels, triglycerides and insulin resistance (HOMA).

## Introduction

Over the past 30 years, obesity has become one of the world’s leading health concerns. A recent report by the OECD observed overweight and obesity to have reached high enough proportions in the industrialized parts of the world to be classified as a global epidemic [1]. The rise in prevalence of obesity and overweight is alarming as high BMI is associated with increased risk of developing complex diseases such as cardiovascular disease, type 2 diabetes, cancer etc. Particularly alarming is the rise in childhood obesity observed in industrialized nations, reaching as high as 16.9% among children in the United States (2008) (http://www.cdc.gov). BMI has a high heritability in twin studies and, during the last two decades, monogenic causes have been determined, e.g. loss of function mutations within the leptin-, and leptin receptor gene, as well as melanocortin 4 receptor (reviewed by Farooqi and O’Rahilly [2]). The genetic influence on weight development is generally more complex however, as has been revealed by genome wide association studies. Large-scale meta-analysis of genome wide association data performed by the GIANT-consortium studies have so far identified at least 32 genetic loci associated with control of body mass development [3]–[4]. While the most powerfully associated obesity-related loci in this study, such as those related to FTO and TMEM18, have been consistently replicated in independent cohorts [5], the effects of other loci have, as of yet, not been consistently reported in independent cohorts. Replication in more specialized cohorts also allows for secondary analysis to discern specific physiological effects of the associated loci or even identification of other associated traits.

**Figure 1:**
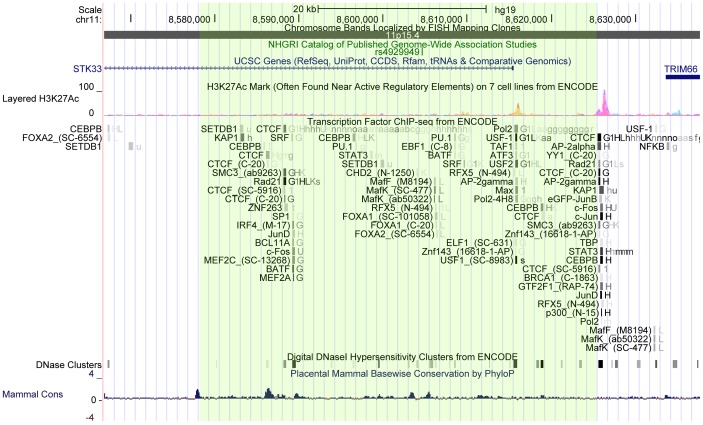
DNA elements within and in the proximity of the rs4929949-related haplotype block. Haplotype blocks were visualized in Haploview according to confidence intervals as described by Gabriel et al. [24]. The haplotype block containing rs4929949 is visualized in green. DNA elements from the Encyclopedia of DNA Elements (ENCODE) were visualized via the UCSC genome browser (htytp://genome.ucsc.edu/). Probable regulatory sites are indicated by acetylation of Lysine 27 on Histone H3 (H3K27ac), DNase sensitivity clusters and chromatin immunoprecipitation sequencing results (ChIP-seq).The haplotype block encompasses one probable regulatory element near the 3′ region of STK33 and lies proximally to an additional upstream regulatory element.

Genetic variants within, and in the proximity of, serine/threonine-protein kinase 33 (STK33) were found to be associated to body weight by the GIANT consortium in 2010 [3]. A single nucleotide polymorphism (SNP), rs4929949, located within intron 1 of STK33, gave the strongest signal in association tests to body mass, but several other SNPs spanning a locus of ∼200 kb, including the entire STK33 gene as well as the proximal upstream area, were also strongly associated [3]. Rs4929949 is a high-frequency SNP with a reported minor allele frequency of about 46% (http://www.1000genomes.org), which makes it suitable for replication in smaller independent cohorts. STK33 is located in a gene-rich region on chromosome 11p15.4 in close proximity to TRIM66 (tripartite motif-containing protein 66), RPL27A (60S ribosomal protein L27a) and ST5 (suppressor of tumorigenicity 5), which are all located within 200 k bp of rs4929949 (Figure 1). Rs4929949 is also located ∼500 kb downstream from the gene encoding TUB (Tubby protein homolog), which has been linked to body weight and obesity in mouse studies [6] as well as early genetic studies [7]–[8], but remains unconfirmed in GWAS. As of yet, no functional data exists linking any of the genes in proximity of rs4929949, or rs4929949 itself, to body weight.

In this study, we investigated the association of rs4929949 to obesity and related phenotypes in two independent European cohorts of children and young adolescents from Sweden and Greece.

## Materials and Methods

### Huddinge Cohort

991 children and adolescents comprising two study groups from the Stockholm area were genotyped for rs4929949 (Table 1). These children comprise a case group of 473 obese children and adolescents enrolled at the National Childhood Obesity Centre at Karolinska University Hospital, as well as 518 healthy Swedish adolescents as controls recruited from secondary schools in the Stockholm area. Healthy controls were recruited via school nurses. Chronically ill, obese or overweight were removed from the controls. Siblings were also removed from analysis to reduce bias. Informed written consent was provided by all participants or by their legal guardians (Table 1). Genomic DNA was extracted from peripheral blood using the QiaGen Maxiprep kit (QiaGen, Hilden, Germany). DNA was stored at −20°C until further processing and genotyping.

**Table 1:** General description of the Swedish and Greek cohorts of children and adolescents.

Greek children		
N (male/female)	2308 (1163/1145)	
Age (years)	11,2±0,7	
Weight (kg)	45,3±11,2	
Height (m)	1,49±0,08	
BMI z-score	0,67±1,00	
**Swedish children and adolescents**	***Control population***	***Obese***
N (male/female)	518 (251/267)	473 (223/250)
Age (years)	17,0±0,9	12,8 −+3,2
Weight (kg)	63,5±10,0	92,9±29,1
Height (m)	1,72±0,09	1,59±0,16
BMI z-score	−0,06±0,72	2,43±0,28

BMI z-scores were calculated according to the LMS method [11] using LMS values from the Center for Disease Control and Prevention (www.cdc.gov).

### Cohort of Greek Children and Adolescents – the Healthy Growth Study

2308 Greek school children and adolescents, 1163 males and 1145 females aged 9–13 years, from a wider region of Athens Greece were genotyped (Table 1). These children were participants of the ‘Healthy Growth Study’, a cross-sectional study on health and behavioral indices of primary schoolchildren living in different parts of Greek territory, initiated in 2007 [9]–[10]. Parents were informed via an extended letter describing the aims of the study. Parental consent was given via a signed consent form. Body weight and height was measured using standard procedures and equipment as part of a school-based health and nutrition education program. BMI-z-score was calculated according to the Obesity Task Force (IOTF) definitions [11]. Stage of pubertal development, i.e. Tanner stage [12], was evaluated by experienced pediatricians via physical examination. Blood samples were obtained after overnight fasting and centrifuged at 3000 rpm for 15 min. Aliquots were stored at −80°C until further processing. Plasma glucose was determined with a enzymatic colorimetric assay (Roche Diagnostics, Visalia, Switzerland). Serum insulin was determined with a chemiluminescence immunoassay (Kyowa Medex Ltd, Minami-Ishiki, Japan).

### Ethical Statement

The study on the Swedish cohort of children and adolescents was approved by the Regional Committee of Ethics, Stockholm (Regionala etikprövningsnämnden, Stockholm). Approval to conduct the study in Greek schools was granted by the Greek Ministry of National Education while ethical approval for the study was granted by the ethics committee of the Harokopio University. Written consent was obtained from all participants or their legal guardians.

### Genotyping

Genotyping of rs4929949 was carried out with a pre-designed Taqman single-nucleotide polymorphism genotyping assay (Applied Biosystems, Foster City, USA) and an ABI7900 genetic analyzer with SDS 2.2 software at the Uppsala Genome Center (http://www.genpat.uu.se/node462). The genotype call rate was 98.3% in the Swedish cohort and 99.7% in the Greek cohort. Tests for deviation from Hardy-Weinberg equilibrium were performed using a Markov chain Monte Carlo test [13] in PLINK v1.07 [14]. No deviations from Hardy-Weinberg equilibrium were detected for rs4929949 in the Swedish and Greek cohorts.

### Statistical Analysis

Statistical analysis was performed in PLINK v1.07 [14]. Logistic regression was used in the cohort of Swedish children to test association of rs4929949 with obesity. Gender effect was included in the model. Linear regression was used to test association of rs4929949 with BMI z-score and phenotypic measurements in the cohort of Greek children; the model was corrected for gender and pubertal development. A p-value <0.05 was considered significant for association of rs4929949 with obesity in the Swedish cohort and BMI z-score in the Greek cohort. Analysis was also performed in PLINK to detect eventual gender-specific effect. We also tested for a recessive model of the effects of rs4929949 in PLINK.

### Power Calculation

Power analysis was performed in CaTS power calculator [15] (http://www.sph.umich.edu/csg/abecasis/CaTS/) and Quanto (http://hydra.usc.edu/gxe/). In the cohort of Swedish adolescents, we had 45% power to detect an association of rs4929949 with obesity at the risk observed in our study at nominal levels of significance (p<0.05), 3% prevalence of childhood obesity in Sweden [16] and assuming an additive model. For the cohort of Greek children we had 72.4% power to detect an association of rs4929949 with BMI at the observed β-value of 0.08147 for the minor allele, assuming an additive model and nominal thresholds for significance (p<0.05).

### Linkage Disequilibrium-analysis

To visualize haplotype blocks co-inherited factors associated with rs4929949, we utilized Haploview 4.2 (www.broadinstitute.org/haploview/haploview) [17] to generate a graphical representation of the linkage disequilibrium (LD) structures from r^2^ scores. HapMap data version 3, release 27 and data from Utah residents with northern and western European ancestry (CEU) combined with “Toscani in Italia” (TSI) was used to generate the linkage disequilibrium (LD) pattern.

## Results

In the Swedish cohort, the minor allele frequency (C-allele) was 50% in obese children and 45.5% in the control population (Table 2). The minor allele frequency in the Greek cohort was 35.8%, which differs from the Swedish cohort and is also somewhat lower than European populations in general (Table 3). This allele frequency is instead more similar to African populations such as Americans of African ancestry in SW USA (ASW) (MAF = 38%, n = 61) and Luhya in Webuye, Kenia (LWK) (MAF = 30%, n = 97), as reported by the 1000 genomes project (http://www.ncbi.nlm.nih.gov/SNP). No deviation from Hardy-Weinberg equilibrium was observed in the Greek cohort, and the observed difference in allele frequency in our study was assumed to be due to natural variation specific to that population.

**Table 2:** Logistic regression analysis shows the minor allele of rs4929949 to be associated with obesity in a cohort of Swedish adolescents diagnosed with obesity.

SNP	Minor allele frequency (C)	Frequency affected	Frequency controls	*p*-value	Odds Ratio (95%CI)
rs4929949	47.64%	50.0%	45.5%	0.047	1.199 (1.002–1.434)
**Genotypes (n)**		**Cases**	**Controls**		
(TT/TC/CC)		151/257/105	115/232/115		

**Table 3:** Linear regression analysis shows the minor allele of rs4929949 to be associated with body weight in a cross-sectional cohort of Greek adolescents.

SNP	Minor allele frequency (C)	*β* (95% CI)	*p*-value
s4929949	35.8%	0.0817 (0.013–0.168)	0.021
**Genotype**	**n**	**BMI z-score (Mean ± SD)**	
TT	930	0.81±1.25	
CT	1094	0.83±1.28	
CC	276	1.02±1.11	

### Rs4929949 is Associated with Clinical Obesity in the Cohort of Swedish Children and with BMI in a Cross-sectional Cohort of Greek Children and Adolescents

**Figure 2:**
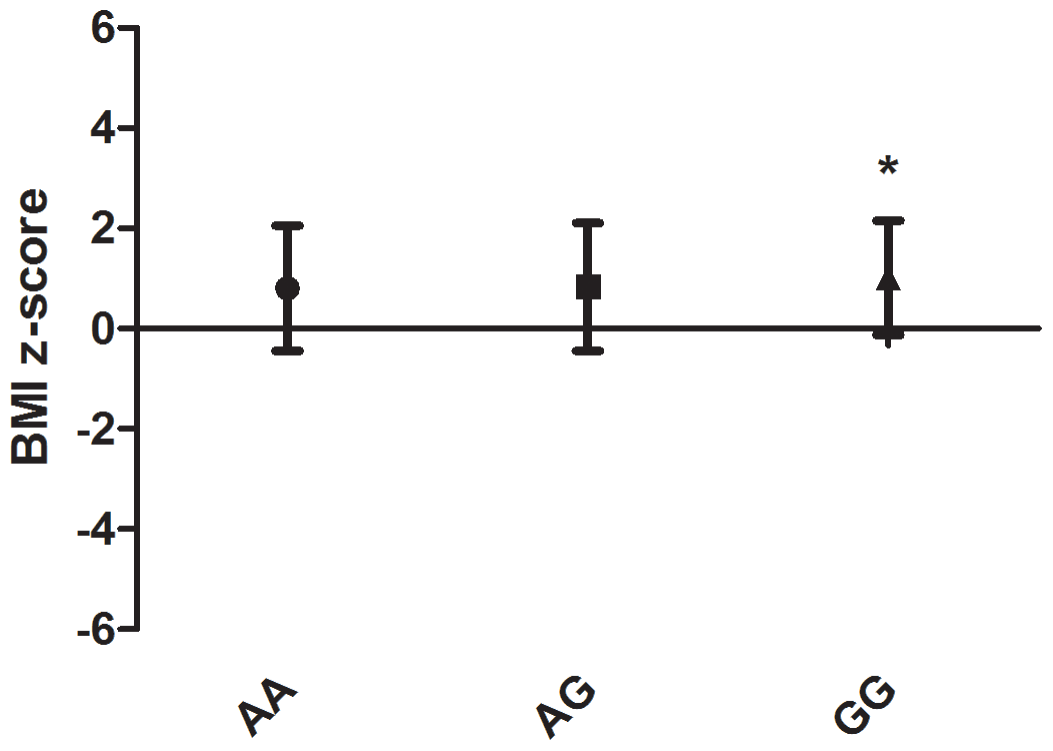
BMI z-scores of the Greek children and adolescents plotted by genotype. Evidence was observed for a recessive model of the effect of rs4929949 on body mass in the cohort of Greek children and adolescents (p = 0.015).

We observe the minor allele of rs4929949 to be associated with obesity in the cohort of children from the Stockholm area (OR = 1.199; 95% CI = 1.002–1.434; p = 0.047) (Table 2). The minor allele of rs4929949 was also observed to be associated with higher BMI in the cohort of Greek children (β = 0.08147; p = 0.033) (Table 3). Analysis of secondary phenotypic measurement like insulin sensitivity (HOMA), daily caloric intake, serum cholesterol, triglycerides and measurements describing body adiposity failed to reveal any associations with rs4929949 (Table S1). Association of rs4929949 with several measurements describing body adiposity were observed at the nominal significance level, as well as a trend towards association with insulin levels which was directionally consistent with non-significant effects reported by Speliotes et al. [3], but only when BMI was not included in the model, indicating these effects to be secondary to the effect of rs4929949 on body weight. No gender-specific effects were observed in either cohort. If we assume a recessive model, we also observe an effect of the minor allele of rs4929949 on body weight in the cohort of Greek children and adolescents (p = 0.015) (Figure 2).

### Rs49299492 is Located in a 47 kb Haplotype Block Encompassing the 3′- and Proximal Upstream Region of STK33

Visualisation of linkage disequilibrium using Haploview reveals rs4929949 to be located within a 47 kb haplotype block spanning from within intron 1 to about 10 kb upstream of STK33 (Figure S1). Visualization of regulatory elements within this region via the encyclopedia of DNA elements (ENCODE), revealed the haplotype block to encompass one probable regulatory site with strong signals of occupancy for upstream regulatory factor 1 (USF-1). This haplotype block is also proximal to a probable regulatory binding site for transcriptional repressor CTCF, CCAAT/enhancer-binding protein beta (CEBPB) and transcription factors c-Jun and Jun-D (Figure 1).

## Discussion

We found that rs4929949 was associated with obesity and body mass development in two independent European cohorts of children and adolescents. The observed effects were directionally consistent with reports from the GIANT consortium. Subsequent analysis of effects of rs4929949 on metabolic factors and body adiposity-related phenotypic measurements failed to reveal any statistically significant association in our cohorts. Effects on body adiposity and insulin levels directionally consistent with reports from the GIANT-consortium [3], were observed when BMI was not included in linear models for association, indicating these effects to be secondary to the effects of rs4929949 on body mass. The effect-sizes that can be observed in our study are of course limited due to the relatively small size of the cohorts. Linkage disequilibrium analysis and ENCODE data revealed one potential regulatory site within the same haplotype block as rs4929949, in the 3′ region of STK33 and one more regulatory site immediately upstream (Figure 1). A possible limitations to our study is the possibility of stratification effects in the Swedish and Greek cohorts as ethnical background was not considered in the recruitment or in the analysis. The recruiting was performed indiscriminately with the aim to reflect the general population and not specific ethnical groups. To minimize potential bias, the control group for the Swedish cohort was recruited from schools in the same region.

Although STK33 has not been directly associated with body mass regulation previously, it is of particular interest because of its potential involvement in GTPase KRas (KRAS) driven cancers and the fact that STK33 has also been targeted pharmacologically. STK33 was first discovered and classified as a serine/threonine-protein kinase putatively related to the Ca^2+^/calmodulin-dependent kinase-family (CAMK) in 2001 [18] and later observed to preferentially be expressed in testes and lung [19] and to target the cytoskeletal protein vimentin for phosphorylation [20]. Although the normal functional role of STK33 has yet to be determined, a synthetic lethality RNA interference-screen identified a dependency between STK33 and the KRAS oncogene [21]. KRAS is one of the most frequently activated oncogenes found in about 17–25% of tumor cells [22]. Despite this high prevalence pharmacological means to inhibit KRAS have yet to emerge. Small molecule inhibition of STK33 *in vitro* did not show sufficient effect on cancer cell-viability, however, leading the authors to speculate on an interaction between mutant KRAS and STK33 independent of its kinase activity [23].

In conclusion, we found that rs4929949 was associated with body mass and obesity in two cohorts of European children and adolescents showing the effects of this locus to be observable already at adolescence. Effects were consistent with previous large scale GWA-studies for body mass reported by the GIANT-consortium [3]. Rs4929949 is located within intron 1 of the gene encoding STK33, a pharmacologically targeted serine/threonine kinase reported to be involved in KRAS-mediated cancers.

## Supporting Information

Figure S1 **Visualization of haplotype blocks within the STK33-encoding region on chromosome 11 of the human genome.** Haploview 4.2 (www.broadinstitute.org/haploview/haploview) was used [17] to generate a graphical representation of the linkage disequilibrium (LD) structures from r^2^ scores. HapMap data version 3, release 27 and data from Utah residents with northern and western European ancestry (CEU) combined with “Toscani in Italia” (TSI) was used to generate the linkage disequilibrium (LD) pattern. The rs4929949-containing haplotype block is highlighted in red.
(PDF)

Table S1 (DOCX)
