# Supplementary figures and images for: The STK33-Linked SNP rs4929949 Is Associated with Obesity and BMI in Two Independent Cohorts of Swedish and Greek Children
Source: PLoS One. 2013 Aug 15;8(8):e71353. doi: 10.1371/journal.pone.0071353 (PMC3744548; doi:10.1371/journal.pone.0071353)

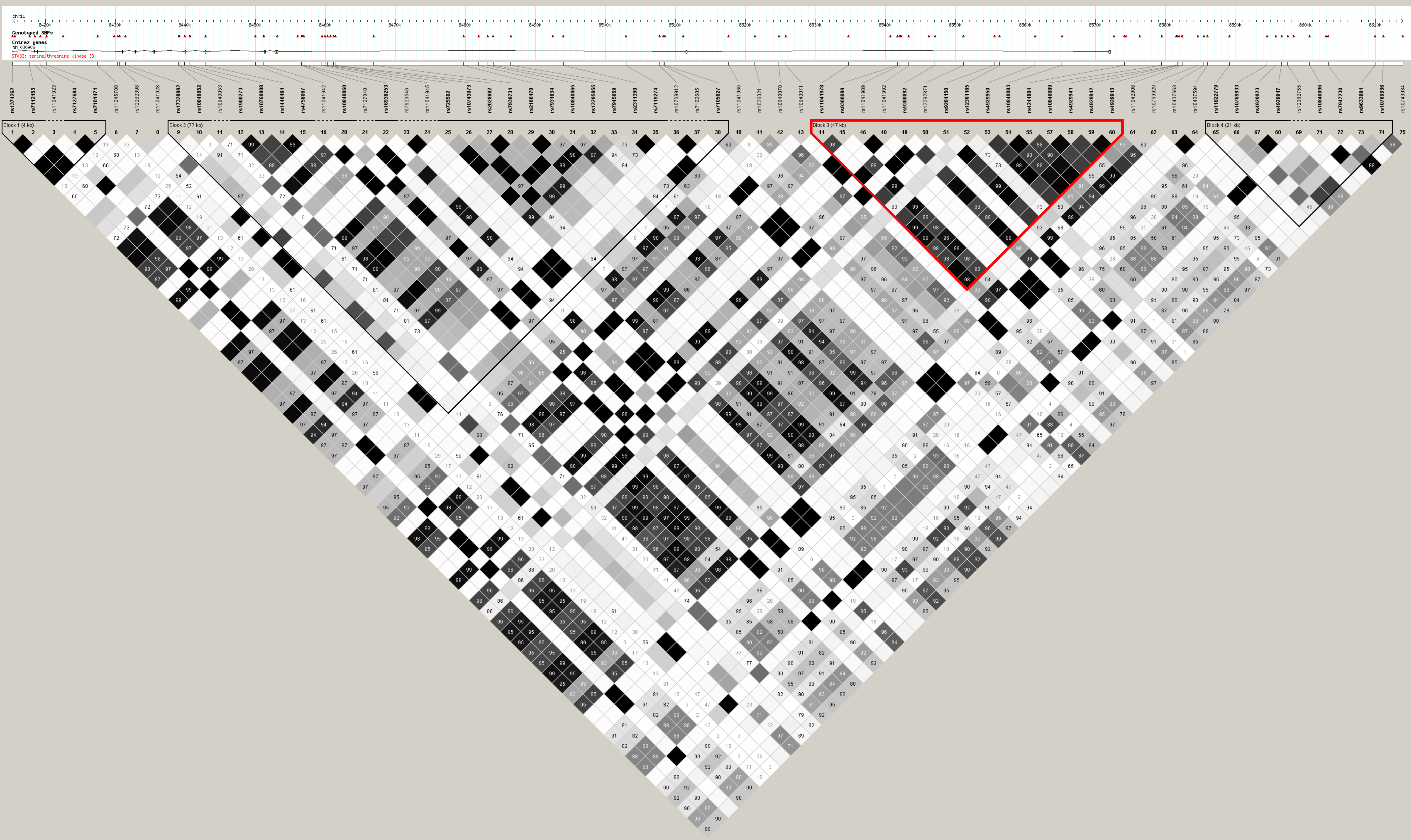

Supplement: Figure S1 — Visualization of haplotype blocks within the STK33-encoding region on chromosome 11 of the human genome. Haploview 4.2 (www.broadinstitute.org/haploview/haploview) was used [17] to generate a graphical representation of the linkage disequilibrium (LD) structures from r2 scores. HapMap data version 3, release 27 and data from Utah residents with northern and western European ancestry (CEU) combined with “Toscani in Italia” (TSI) was used to generate the linkage disequilibrium (LD) pattern. The rs4929949-containing haplotype block is highlighted in red. (PDF) [file pone.0071353.s001.pdf]
