# Supplementary material for: The STK33-Linked SNP rs4929949 Is Associated with Obesity and BMI in Two Independent Cohorts of Swedish and Greek Children
Source: PLoS One. 2013 Aug 15;8(8):e71353. doi: 10.1371/journal.pone.0071353 (PMC3744548; doi:10.1371/journal.pone.0071353)
Supplement: Table S1 — (DOCX) [file pone.0071353.s002.docx]

| **Metabolic traits** | Informative data (n) | *β* | *t*-statistic | *p*-value |
| --- | --- | --- | --- | --- |
| Fasting plasma glucose (mM) | 1998 | 2.64E-03 | 0.15 | 0.88 |
| Plasma Insulin (mM) | 2259 | 1.67E-06 | 1.18 | 0.24 |
| HOMA | 1984 | 3.38E-03 | 0.45 | 0.65 |
| HDL cholesterol (mg/dl) | 2265 | -5,88E-02 | -0.13 | 0.89 |
| LDL cholesterol | 2265 | -5.19E-01 | -0.61 | 0.54 |
| Total cholesterol (mg/dl) | 2265 | -4.45E-01 | -0.44 | 0.66 |
| triglycerides (mg/dl) | 2265 | 6.67E-01 | 0.78 | 0.43 |
| **Adiposity measurements** |  |  |  |  |
| waist-hip ratio | 2279 | 1.17E-03 | 0.69 | 0.49 |
| Average biceps skinfold thickness | 2280 | -1.61E-03 | -0.37 | 0.72 |
| Avegare subscapular skinfold thickness | 2280 | 2.76E-03 | 0.70 | 0.49 |
| Avegare suprailliac skinfold thickness | 2280 | 3.22E-03 | 0.68 | 0.49 |
| Average triceps skinfold thickness | 2280 | 1.82E-01 | 1.43 | 0.15 |
| **Average dietary energy intake (Kcal/day)** | 2261 | -3.35E-03 | -0.82 | 0.41 |
|  |  |  |  |  |

**Table S1.** Results from linear regression analysis for association of rs4929949 with phenotypic traits describing metabolic factors, body adiposity and eating behavior. Models were co-varied for pubertal development (Tanner stage), age, gender, and BMI z-score. Variable-distribution was examined using MiniTab Release 14.20. Non-normally distributed variables were normalized through logarithmization. Linear regression analysis was performed in PLINK v1.07.
